# The Mechanisms of Action of Ribosome-Targeting Peptide Antibiotics

**DOI:** 10.3389/fmolb.2018.00048

**Published:** 2018-05-14

**Authors:** Yury S. Polikanov, Nikolay A. Aleksashin, Bertrand Beckert, Daniel N. Wilson

**Affiliations:** ^1^Department of Biological Sciences, University of Illinois at Chicago, Chicago, IL, United States; ^2^Department of Medicinal Chemistry and Pharmacognosy, University of Illinois at Chicago, Chicago, IL, United States; ^3^Center for Biomolecular Sciences, University of Illinois at Chicago, Chicago, IL, United States; ^4^Institute for Biochemistry and Molecular Biology, University of Hamburg, Hamburg, Germany

**Keywords:** proline-rich antimicrobial peptides, ribosome, translation, inhibitor, antibiotic

## Abstract

The ribosome is one of the major targets in the cell for clinically used antibiotics. However, the increase in multidrug resistant bacteria is rapidly reducing the effectiveness of our current arsenal of ribosome-targeting antibiotics, highlighting the need for the discovery of compounds with new scaffolds that bind to novel sites on the ribosome. One possible avenue for the development of new antimicrobial agents is by characterization and optimization of ribosome-targeting peptide antibiotics. Biochemical and structural data on ribosome-targeting peptide antibiotics illustrates the large diversity of scaffolds, binding interactions with the ribosome as well as mechanism of action to inhibit translation. The availability of high-resolution structures of ribosomes in complex with peptide antibiotics opens the way to structure-based design of these compounds as novel antimicrobial agents.

## The ribosome and translation as an antibiotic target

**Figure 1:**
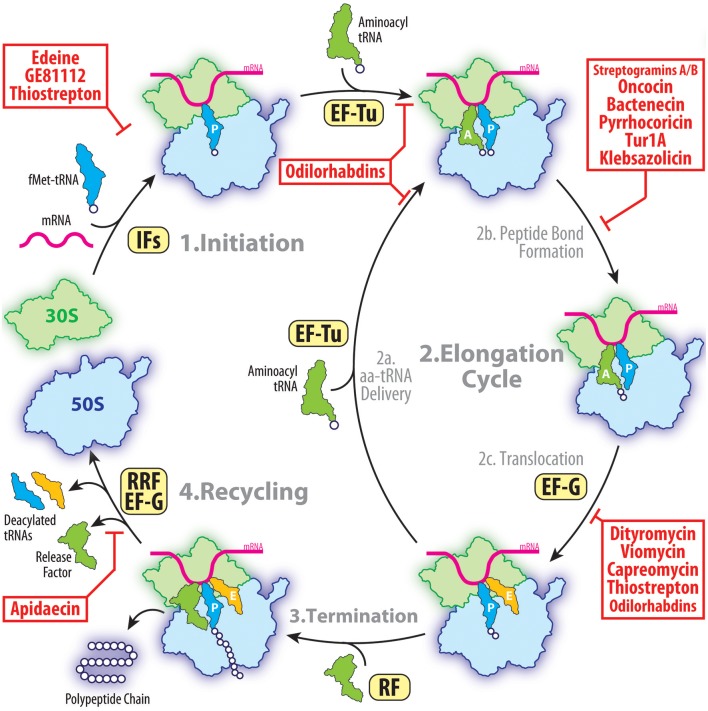
The target of peptides antibiotics during the proteins synthesis cycle. The initiation of the translation involves the binding of the initiator fMet-tRNA and mRNA to form a 70S pre-initiation complex with the fMet-tRNA located at the P site. This process is facilitated by initiation factors (IFs) and is inhibited by peptide antibiotics edeine, GE81112 and thiostrepton. During elongation, the aminoacyl-tRNAs are delivered to the A site by the elongation factor Tu (EF-Tu) allowing subsequent peptide bond formation to occur. This step of translation can be inhibited by streptogramins A/B, oncocin-112, bactenecin-7, or klebsazolicin. Following peptide bond formation, the tRNAs are translocated through the ribosome by the elongation factor G (EF-G). This step of elongation is inhibited by dityromycin, tuberactinomycins, or thiostrepton. After multiple elongation cycles, one of the three stop codons appears in the A site of the ribosome and release factors (RFs) are typically recruited. Apidaecin specifically inhibits the termination process by preventing the RFs from dissociating from the ribosome. Following polypeptide release, the post-termination ribosome is recycled by the ribosome recycling factor (RRF) and EF-G so that the components can be reused for the next round of translation.

The ribosome is one of the most conserved and sophisticated macromolecular machines of the cell. It is composed of two unequal subunits, a small 30S and large 50S in bacteria, which join together to form a 70S ribosome. While each ribosomal subunit contains a large number of ribosomal proteins, it is the ribosomal RNA (rRNA) that plays the most critical functional role defining the ribosome as a ribozyme (Nissen et al., 2000). The small subunit decodes the genetic information delivered by messenger RNA (mRNA), whereas the large subunit hosts the catalytic peptidyl transferase center (PTC), where amino acids delivered by transfer RNAs (tRNAs) are linked into polypeptides (reviewed in Arenz and Wilson, 2016). The ribosome provides a platform for binding of the mRNA and transfer RNAs (tRNAs). The tRNAs have two functional ends, one carrying the amino acid and the other end containing the anticodon that recognizes the codon of the mRNA. The ribosome has three tRNA binding sites: the aminoacyl (A), peptidyl (P), and exit (E) sites. The A site binds the incoming aminoacyl-tRNA (aa-tRNA), the P site binds the peptidyl-tRNA carrying the nascent polypeptide chain and the E site binds deacylated tRNA before it dissociates from the ribosome. For translation to proceed efficiently, many protein factors are needed, which sequentially guide the ribosome through the protein synthesis cycle (Figure 1). Translation is initiated on the 30S subunit with the help of initiation factors that recruit the initiator formyl-methionine tRNA (fMet-${\text{tRNA}}_{\text{i}}^{\text{fMet}}$) to the ribosomal P site where it recognizes the start codon of the mRNA. The 50S subunit associates with the 30S, forming the 70S initiation complex that is primed for the elongation phase of protein synthesis. The second codon of the open reading frame located in the A site of the ribosome is decoded by the ternary complex, composed of aa-tRNA, elongation factor Tu (EF-Tu), and GTP. Decoding of the A-site codon by a cognate aa-tRNA triggers GTP hydrolysis on EF-Tu and release of the aa-tRNA into the A site. The CCA-3′ terminus of aa-tRNA can then accommodate into the PTC of the 50S subunit, and the peptidyl transferase reaction occurs spontaneously extending the nascent peptide chain by one amino acid residue. As the polypeptide is synthesized it passes through a tunnel on the large ribosomal subunit. The function of this exit tunnel appears to be not only to provide an unobstructed passage through the ribosome for newly synthesized polypeptide chains but in many cases to regulate translation itself. Specific elements within the tunnel monitor the amino acid sequence of the nascent polypeptide chain and can arrest translation in response to particular co-factors, such as drugs or metabolites (Ito and Chiba, 2013; Wilson et al., 2016). Following peptide bond formation, translocation of mRNA and tRNAs is catalyzed by the elongation factor EF-G. Translocation by EF-G shifts the deacylated tRNA from the P site to the E site and the peptidyl-tRNA from the A site to the P site. The elongation cycle of EF-Tu delivery of aa-tRNAs and subsequent translation by EF-G is repeated until a stop codon enters the A site. Release factors (RFs), such as RF1 and RF2, recognize the stop codon and promote hydrolysis of the peptidyl-tRNA in the P site, releasing the newly synthesized protein from the ribosome. The 70S ribosome is then recycled into individual subunits by the concerted action of EF-G and the ribosome recycling factor (RRF; Figure 1).

There is a diverse range of clinically important antibiotics that interfere with protein synthesis by binding at various functional centers of the ribosome and either freezing a particular conformation of the ribosome or hindering the binding of its ligands (Wilson, 2009, 2014). Although these antibiotics have been successfully employed during the past 70 years for the treatment of infectious diseases, the rapid spread of antibiotic resistance among pathogenic microorganisms has greatly limited the medical utility of our existing antibiotic arsenal. This poses a serious healthcare threat, highlighting the urgent need for new classes of compounds and/or improvement of existing antibiotics. The increase in multi-drug resistant pathogens has stimulated the development of new approaches to revive the natural products discovery pipeline and to enrich our treasure trove of structural scaffolds suitable for optimization by medicinal chemists. One such avenue is the discovery and optimization of peptide-based antibiotics. Peptide antibiotics provide an unmatched platform for rational drug design because most of them can be chemically synthesized. This allows the peptide antibiotics to be easily altered by simply changing the primary sequence of amino acids as well as incorporating non-natural amino acids and chemical moieties. The many natural product peptide antibiotics that have already been discovered usually fall into one of three classes: (i) ribosomally-synthesized peptides, such as proline-rich antimicrobial peptides (PrAMPs); (ii) ribosomally-synthesized and post-translationally modified peptides (RiPPs), such as klebsazolicin (KLB) and thiopeptides (thiostrepton, micrococcin); or (iii) peptides produced by non-ribosomal peptide synthetases (NRPSs), such as edeine and GE81112. With the exception of the streptogramins, none of the natural product peptide antibiotics that have been identified and characterized have so far been used clinically, however, the recent structures of these peptide antibiotics on the ribosome provides the opportunity to further develop these classes of potent antimicrobial agents. Here we provide an overview on the known ribosome-targeting peptide antibiotics that have been biochemically and structurally characterized. The nine different classes are organized in the following sections based on whether they target the small or large subunit of the ribosome.

## Peptide antibiotics targeting the small ribosomal subunit

**Figure 2:**
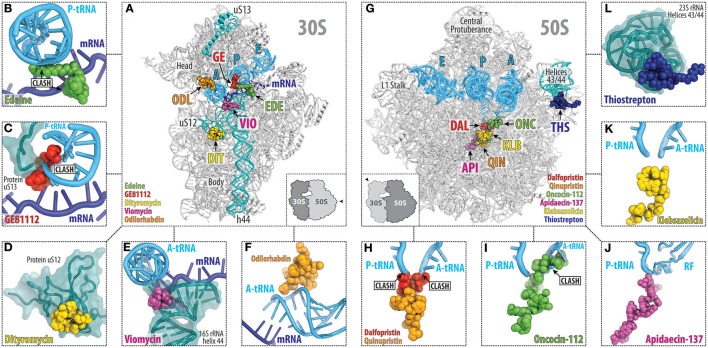
Overview of the peptide antibiotics binding sites on the bacterial ribosome. Overview **(A)** and close-up views **(B–F)** of the binding sites of the peptide antibiotics **(B)** edeine B (EDE, green), **(C)** GE81112 (GE, red), **(D)** dityromycin (DIT, yellow), **(E)** viomycin (VIO, magenta), and **(F)** odilorhabdin (ODL, orange), which target the small (30S) ribosomal subunit. The mRNA (blue) and anticodon stem loop (ASL) of A-, P-, and E-site tRNAs (cyan) are shown, and 16S rRNA helix h44 as well as ribosomal proteins uS12 and uS13 are highlighted for reference. Overview **(G)** and close-up views **(H–L)** of the binding sites of the peptide antibiotics **(H)** streptogramin type A (dalfoprsitin, DAL, red) and type B (quinupristin, QIN, orange), **(I)** oncocin-112 (ONC, green), **(J)** apidaecin-137 (API, magenta), **(K)** klebsazolicin (KLB, yellow), and **(L)** thiostrepton (THS, blue), which target the large (50S) ribosomal subunit. The relative position of A, P, and E-site tRNAs (cyan) are shown, and 23S rRNA helices H43/44 is highlighted for reference.

So far there are five main classes of peptide antibiotics that target the small ribosomal subunit (Figures 2A–F), two of which, target translation initiation, for example, edeine and GE81112 (Figure 1), whereas the other three, the dityromycin/GE82832, the tuberactinomycin (viomycin and capreomycin), and odilorhabdin families, inhibit the translocation and/or decoding step (Figures 1, 2A–F). While edeine, GE81112, odilorhabdins, and tuberactinomycins interact predominantly with the 16S rRNA to modulate tRNA binding (Figures 2B,C,E,F; Pioletti et al., 2001; Stanley et al., 2010; Fabbretti et al., 2016), dityromycin/GE82832 interact with ribosomal protein uS12 (Figure 2D) to inhibit translocation by trapping EF-G in a compact conformation on the ribosome (Bulkley et al., 2014; Lin et al., 2015).

### Edeine inhibits initiation complex formation

**Figure 3:**
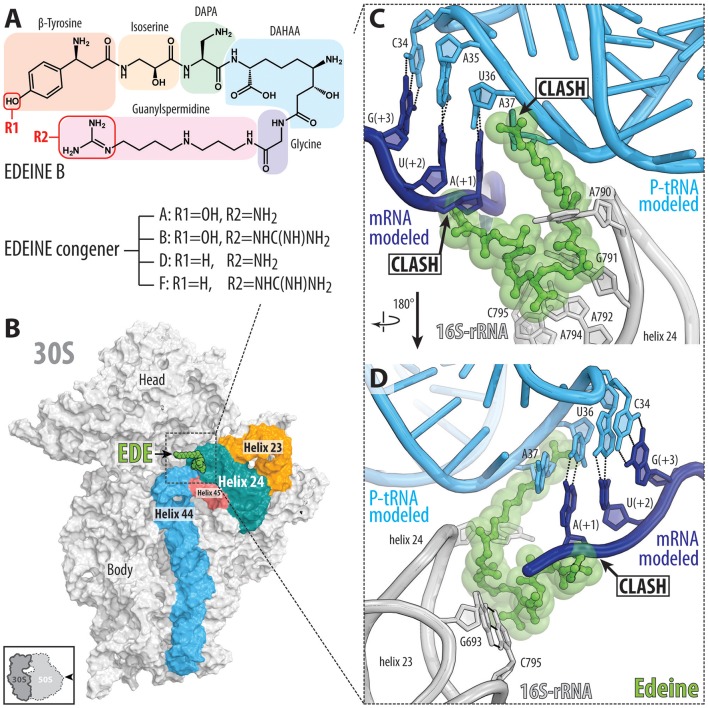
Binding of the peptide antibiotic edeine is incompatible with the P-site tRNA and mRNA. **(A)** Chemical structure of the edeine B consisting of β-tyrosine, isoserine, DAPA (2,3-diaminopropanoic acid), DAHAA (2,6-diamino-7-hydroxyazelaic acid), and guanylspermidine moities. **(B)** Overview of edeine B (EDE, green) binding site on the 30S subunit (PDB ID 1I95; Pioletti et al., 2001), with 16S rRNA helices h44 (blue), h45 (red), h23 (orange), and h24 (teal) shown for reference. The 30S subunit is viewed from the subunit interface as indicated by the inset at the bottom left. **(C,D)** Close-up view of EDE (green) binding site at the tip of helix h23 and h24 (gray) showing overlap of EDE with P-site tRNA (cyan) and first codon (+1) of the P-site mRNA (blue). Hydrogen bonding between the nucleotides G693-C795 of the 16S rRNA formed upon EDE binding is indicated with dashed lines in **(D)** (Pioletti et al., 2001).

The edeine (EDE) class of antibiotics are pentapeptide amide antibiotics produced by the bacterium *Bacillus brevis* Vm4 (Gale et al., 1981). For example, the active isomer of edeine B has an N-terminal β-tyrosine residue linked to a C-terminal guanylspermidine moiety via glycine and three non-proteinogenic amino acids, 2,3-diaminopropanoic acid (DAPA), 2,6-diamino-7-hydroxyazelaic acid (DAHAA), and isoserine (Figure 3A; Westman et al., 2013). Edeines display activity against both Gram-positive and -negative bacteria, and also *Mycoplasma* sp. (Gale et al., 1981). X-ray structures reveal that EDE has a single binding site on the small 30S subunit, positioned on the solvent side of the platform, spanning between helices h24, h44, and h45 (Pioletti et al., 2001; Figure 3B). The guanylspermidine moiety of EDE overlaps with the position of the anticodon stem loop of a P-site tRNA (Figure 3C; Pioletti et al., 2001), consistent with the inhibition of binding of initiator tRNA to the P site of 30S subunits and 70S ribosomes (Dinos et al., 2004). Curiously, however, EDE does not inhibit binding of aa-tRNAs to the P site of 70S ribosomes in the absence of mRNA, leading to the suggestion that EDE may influence binding of the P-site tRNA indirectly *via* perturbing the path of the mRNA (Dinos et al., 2004). Binding of EDE induces base-pair formation between G693 and C795 (*E. coli* numbering is used throughout the text) at the tips of h23 and h24, respectively (Figure 3D; Pioletti et al., 2001), in agreement with the observation that EDE protects these nucleotides from chemical modification (Woodcock et al., 1991). The G693-C795 base-pair induced by EDE appears to obstruct the path of the mRNA and may therefore explain the indirect effect that EDE has on P-site tRNA binding. Whether direct or indirect, by blocking binding of the initiator tRNA to the 30S subunit, EDE inhibits formation of the 30S pre-initiation complex and thereby blocks association of the large subunit to form a competent 70S initiation complex.

EDE has also been shown to inhibit translation initiation on eukaryotic cytoplasmic ribosomes, such as in yeast (Gale et al., 1981). A recent crystal structure of the yeast 80S ribosome in complex with EDE reveals that although the binding site overlaps with that observed in bacteria, it adopts a markedly different conformation on the ribosome (Garreau de Loubresse et al., 2014). Rather than encroaching onto the P site as on the bacterial small ribosomal subunit, EDE is bound exclusively in the E site of the yeast small subunit (Garreau de Loubresse et al., 2014). Nevertheless, in yeast, EDE appears to also preclude stable binding of the initiator tRNA at the P site, which leads to continuous scanning of yeast 40S subunits (Kozak and Shatkin, 1978).

### GE81112 targets translation initiation

**Figure 4:**
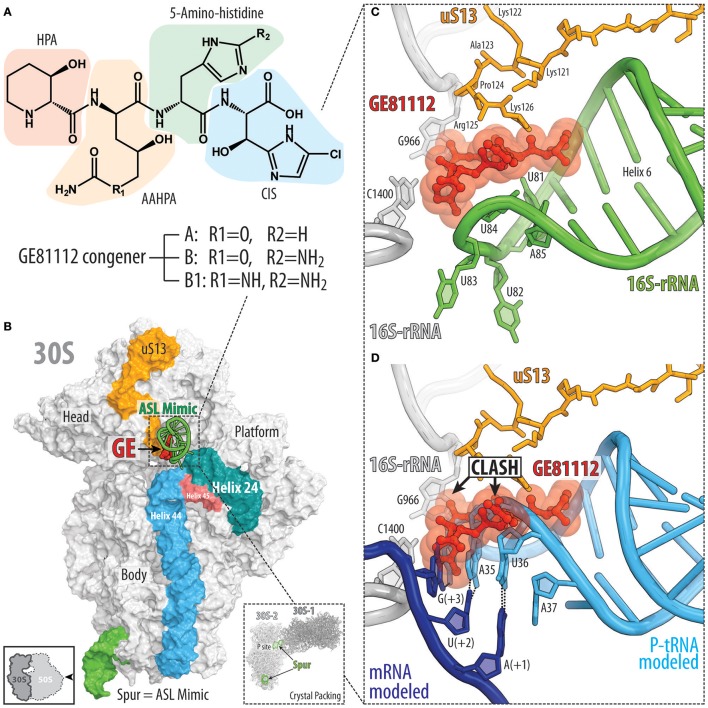
Binding site of GE81112 on the 30S subunit. **(A)** Chemical structure of GE81112 congeners A, B, and B1. HPA—3-hydroxypipecolic acid; AAHPA—2-amino-5-[(aminocarbonyl)oxy]-4-hydroxypentanoic acid; CIS—5-chloro-2-imidazolylserine. **(B)** Overview of GE81112 binding site on the 30S subunit (PDB ID 5IWA; Fabbretti et al., 2016), with 16S rRNA helices h44 (cyan), h45 (red), and h24 (teal) as well as ribosomal protein uS13 (orange) and anticodon stem loop (ASL) mimic (green) of the P-site tRNA shown for reference. The 30S subunit is viewed from the subunit interface as indicated by the inset at the bottom left. The inset on the bottom right shows packing of the 30S ribosomal subunits in the crystal. Note that the spur (green) of one 30S subunit (30S-1, dark gray) inserts into the P site of the other 30S subunit (30S-2, light gray) and mimics ASL of the P-site tRNA. **(C)** Close-up view of the binding site of GE81112 within the ASL mimic (spur, helix 6, green) compared with **(D)** canonical binding position and conformation of the ASL of a P-site tRNA (cyan) and mRNA (blue).

The GE81112 family of non-ribosomally synthesized tetrapeptide antibiotics are produced by some *Streptomyces* species (Brandi et al., 2006a,b). The GE81112 biosynthetic gene cluster (*getA-N*) has been identified in *Streptomyces* sp. L-49973, leading to a linear model for GE81112 synthesis via a series of non-ribosomal peptide synthetases (NRPSs) and non-NRPS enzymes (Binz et al., 2010). GE81112 peptides are comprised of four L-amino acids: 3-hydroxypipecolic acid (HPA), 2-amino-5-[(aminocarbonyl)oxy]-4-hydroxypentanoic acid (AAHPA) followed by 5-amino-histidine and 5-chloro-2-imidazolylserine (CIS) residues (Figure 4A; Brandi et al., 2006a,b). Three distinct GE81112 congeners (A, B, and B1) have been identified, differing in molecular mass between 643 and 658 Da, with the most active and best studied being the B1 variant (658 Da; Figure 4A). GE81112 displays excellent activity against a variety of Gram-positive and Gram-negative bacteria (Brandi et al., 2006c; Maio et al., 2016). However, in rich media (e.g., LB broth), the inhibitory effects of GE81112 are supressed because the uptake of GE81112 occurs via the oligopeptide permease Opp, which is blocked by the excess of various peptides present in the media competing with GE81112 for Opp binding (Brandi et al., 2006c; Maio et al., 2016). Indeed, the majority of spontaneous resistance mutations that arise in bacteria exposed to GE81112 lead to inactivation of the Opp transporter (Maio et al., 2016).

GE81112 was originally discovered in a high-throughput screen of Actinomycetes secondary metabolites that display inhibitory activity in an *E. coli in vitro* cell-free translation system, but not in a yeast system (Brandi et al., 2006a,b). To specifically select for novel translation initiation inhibitors, the screen was performed using two different mRNAs, a natural mRNA that is dependent on canonical translation initiation and a synthetic poly(U) mRNA that does not require canonical initiation events for translation to occur. The inhibition of translation *in vitro* by GE81112 from the screen was validated *in vivo* by showing that GE81112 inhibits the incorporation of radiolabeled [^14^C]-phenylalanine, but not [^3^H]-thymidine or [^3^H]-uridine, thus, confirming GE81112 to be an inhibitor of protein synthesis, but not of DNA replication or RNA transcription (Brandi et al., 2006a,b). Subsequent experiments revealed that GE81112 does not inhibit *in vitro* translation when using a human (HeLa) system, but is active in an archaeal (*Sulfolobus sulfataricus*) system (Brandi et al., 2006a,b) pointing to its selectivity against prokaryotic translation.

Initial biochemical assays suggested that GE81112 inhibited formation of the 30S pre-initiation complex (30S-PIC) by preventing binding of the initiator fMet-tRNA to the 30S subunit (Brandi et al., 2006b). However, this model was subsequently revised, such that GE81112 does not interfere with the initial binding of the fMet-tRNA in the “unlocked” 30S-PIC, but prevents conversion of the “unlocked” into the “locked” 30S-PIC (Fabbretti et al., 2016; Lopez-Alonso et al., 2017). Correct recognition of the start codon by the fMet-tRNA is thought to facilitate conversion from the “unlocked” to the “locked” 30S-PIC, which is accompanied by conformational changes in the ribosome and fMet-tRNA that promote joining of the 50S subunit to form the 70S initiation complex. Cryo-electron microscopy structures of the 30S-PIC formed in the presence of GE81112 revealed two distinct functional ribosomal states with the fMet-tRNA either directly engaged with the start codon, or shifted away and disengaged from the mRNA (Lopez-Alonso et al., 2017). Unfortunately, the resolution of the complexes did not allow visualization of GE81112 so it remains unclear whether both states represent unlocked 30S-PIC with GE81112 bound or whether the engaged state reflects the locked 30S-PIC in the absence of GE81112.

The structure of GE81112 on the *Thermus thermophilus* 30S subunit was determined using X-ray crystallography (Fabbretti et al., 2016). GE81112 was found to bind to helix 6 (h6) of the 16S rRNA, which forms the so-called spur of the 30S subunit (Figure 4B). Within the crystal, the individual 30S subunits are packed in such a way that the spur of one 30S subunit mimics the anticodon-stem-loop (ASL) of a tRNA and inserts into the P site of another 30S subunit, suggesting that GE81112 binds and interacts with the ASL of a P-site tRNA in the 30S-PIC (Figure 4C; Fabbretti et al., 2016). Binding of GE81112 to the 30S subunit induces distortions within the loop of h9, which mimics the anticodon of the P-site tRNA (Figures 4C,D), suggesting how GE81112 could prevent recognition of the start codon by the P-site tRNA. In addition to h9, GE81112 establishes extensive contact with the C-terminal extension of the ribosomal protein uS13 (Figures 4C,D). This interaction is, however, unlikely to be necessary for the action of GE81112 since it cannot occur in many other bacterial species, such as *E. coli*, because the C-terminal extension of the uS13 is significantly shorter.

Other than uS13, very few additional contacts of GE81112 are observed with the 30S subunit, suggesting that the P-site tRNA comprises the major determinant for GE81112 binding. The extensive interaction with P-site tRNA, rather than with the 16S rRNA, may explain the difficulty in obtaining spontaneous resistance mutations to GE81112, even when the Opp transporter is overexpressed (Maio et al., 2016). However, 16S rRNA mutations A794G/U or G926A/C/U that mediate high level (up to 70-fold) kasugamycin resistance, also lead to a modest increase (10-fold) in GE81112 resistance, as monitored using *in vitro* mRNA translation assays (Maio et al., 2016). Similarly, the extensive interaction with P-site tRNA, rather than with 16S rRNA, may also explain the difficulty in using chemical modification techniques to map the GE81112 binding site on the 30S subunit (Brandi et al., 2006b). Nevertheless, chemical probing experiments revealed that binding of GE81112 induces conformational changes within the h44/h45/h24a interface of the 30S subunit (Brandi et al., 2006b; Fabbretti et al., 2016), which were proposed to favor the disengaged conformation of the initiator tRNA and prevent conversion to the “locked” 30S-PIC and thereby prevent 50S subunit joining (Fabbretti et al., 2016).

### GE82832/dityromycin targets the translocation step of translation elongation

**Figure 5:**
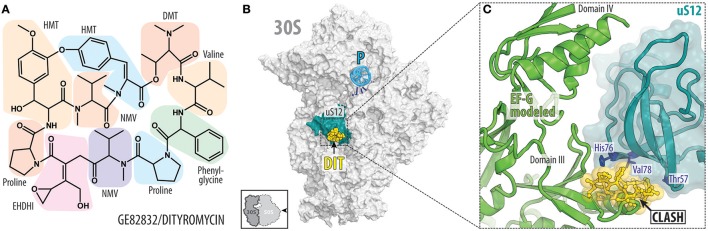
GE82832/dityromycin bind to uS12 on the 30S subunit and inhibit translocation. **(A)** Chemical structure of the GE82832/dityromycin comprises proteinogenic (e.g., proline or valine) as well as non-proteinogenic amino acids, such as N,N-dimethyl-threonine (DMT), N-methyl-valine (NMV), epoxy-hydroxy-dehydro-isoleucine (EHDHI), or dihydroxyl-methyl tyrosine (HMT). **(B)** GE82832/dityromycin (DIT, yellow) interacts exclusively with the ribosomal protein uS12 (teal) on the 30S subunit (gray) (PDB ID 4NVY; Bulkley et al., 2014). The anticodon stem loop (ASL) of a P-site tRNA (cyan) and mRNA (blue) are shown for reference. The 30S subunit is viewed from the subunit interface, as indicated by the inset at the bottom left. **(C)** Overlap in the binding site of dityromycin (yellow) and domain III of EF-G (green). Residues within uS12 (teal) that are important for dityromycin binding are highlighted in blue.

While ribosomal protein uS13 contributes significantly to the binding of GE81112, ribosomal protein uS12 in the 30S subunit is the important determinant for binding of GE82832, a cyclic peptide antibiotic (Figure 5A) produced by *Streptosporangium cinnabarinum* (strain GE82832) that inhibits tRNA translocation by interacting with the 30S subunit (Brandi et al., 2006a). GE82832 is related to a poorly characterized antibiotic dityromycin that was discovered decades ago (Omura et al., 1977; Brandi et al., 2012). Characterization of both antibiotics has shown that they are structurally and functionally related, with both inhibiting EF-G-dependent tRNA translocation on the ribosome (Brandi et al., 2012). The crystal structure of the 70S ribosome in complex with dityromycin and GE82832 showed that these antibiotics are unique because they bind exclusively to ribosomal protein (uS12) rather than rRNA (Figures 5B,C; Bulkley et al., 2014). uS12 is positioned on the shoulder of the 30S subunit, where it reaches into the decoding center and acts as a control element in tRNA selection (Yates, 1979) and the translocation of tRNA-mRNA through the ribosome (Cukras et al., 2003).

The mechanism by which dityromycin and GE82832 interfere with tRNA and mRNA translocation has recently been elucidated using a crystal structure of EF-G bound to a dityromycin-70S ribosome complex (Figure 5C; Lin et al., 2015). The binding of dityromycin to protein uS12 traps EF-G in a compact conformation on the ribosome, inhibiting EF-G-mediated tRNA translocation. The binding site of GE82832/dityromycin also overlaps with that of ribosome recycling factor (RRF; Gao et al., 2007). Because RRF and EF-G work together in recycling, it is unclear whether the effects of GE82832/dityromycin on RRF could be disentangled from its effects on EF-G alone, but a superposition of RRF bound to both the *E. coli* Borovinskaya et al., 2007 and *T. thermophilus* (Weixlbaumer et al., 2007) ribosomes shows that RRF and GE82832/dityromycin share a contact point with uS12.

While the structure of GE82832/dityromycin in complex with the bacterial ribosome and EF-G explains its activity as a translocation inhibitor, it is also consistent with GE82832/dityromycin affecting the ability of EF-Tu to deliver aminoacyl-tRNA to the ribosomal A-site. The mutation of several residues of uS12 that are distant from the decoding center have been shown to increase miscoding errors. Two of these mutations, Thr57 and Val78 (*E. coli*; Agarwal et al., 2011), form part of the binding pocket for GE82832/dityromycin. Moreover, His76, the same residue that is critical for binding of GE82832/dityromycin to the ribosome (Brandi et al., 2012), is involved in EF-Tu signaling when codon recognition has taken place (Gregory et al., 2009). However, only at high concentrations (~10 μM) does GE82832/dityromycin inhibit (~50%) the delivery of tRNA to the A-site in the absence of EF-Tu, whereas it has virtually no effect when EF-Tu is present (Brandi et al., 2006a). While this is likely due to the fact that aa-tRNA and EF-Tu simply outcompete the antibiotic from its binding site, it should be noted that overall protein synthesis and translocation are inhibited at the same rate by GE82832/dityromycin (Brandi et al., 2006a).

### The tuberactinomycins viomycin and capreomycin inhibit translocation

**Figure 6:**
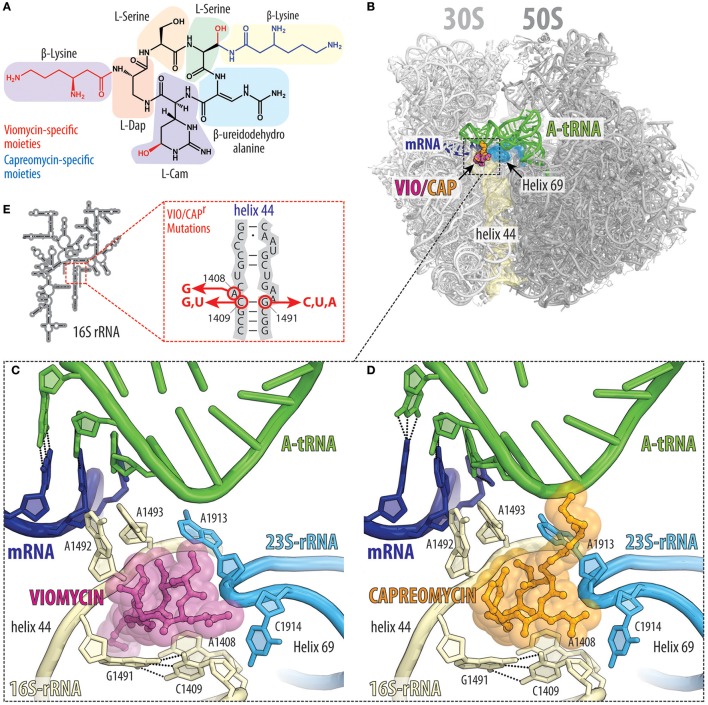
Tuberactinomycins bind to the intersubunit bridge to inhibit translocation. **(A)** Chemical structures of the tuberactinomycins viomycin and capreomycin, with the chemical core (black) and drug-specific moieties colored red (viomycin) or blue (capreomycin). Tuberactinomycins are comprised of both proteinogenics (e.g., serine) as well as non-proteinogenic amino acids [e.g., (2S,3R)-capreomycidine (L-Cam), or L-2,3-diaminopropionic acid, L-Dap]. **(B)** Overview and **(C,D)** close-up views of the **(C)** viomycin (VIO, magenta), and (**D**) capreomycin (CAP, orange) binding sites (PDB IDs 4V7H and 4V7M, respectively; Stanley et al., 2010), both of which are located between helix h44 (yellow) on the 30S subunit and helix H69 (cyan) on the 50S subunit. Tuberactinomycin binding induces nucleotides A1492 and A1493 of the 16S rRNA to flip out of helix h44 and interact with the mRNA (blue) and A-site tRNA (green) duplex that is formed during decoding. **(E)** Secondary structure of the 16S rRNA and positions of the resistance mutation within helix h44.

Viomycin and capreomycin are cyclic pentapeptide antibiotics containing several non-canonical amino acids (Figure 6A), which are produced by non-ribosomal peptide synthetases (NRPSs) found in various *Streptomyces* species (Thomas et al., 2003). Viomycin and capreomycin are members of the tuberactinomycin family and display excellent activity against *Mycobacterium tuberculosis*, including multidrug resistant strains (Jain and Dixit, 2008). Tuberactinomycins have a single binding site on the ribosome that spans the ribosomal interface between h44 of the small 30S subunit and H69 of the large 50S subunit (Figure 6B; Stanley et al., 2010). Binding of the tuberactinomycins within h44 requires nucleotides A1492 and A1493 to adopt a flipped-out conformation (Figures 6C,D; Stanley et al., 2010), as observed during decoding of aa-tRNA in the A-site (Ogle et al., 2003). This explains why the affinity of viomycin to the ribosome greatly increases upon binding of an A-site tRNA (Holm et al., 2016). Although the crystal structures of viomycin (and capreomycin) were on non-rotated ribosomes, biophysical studies indicate that viomycin stabilizes a rotated conformation of the ribosome with hybrid A/P- and P/E-tRNAs (Peske et al., 2004; Shoji et al., 2006; Ermolenko et al., 2007; Pan et al., 2007; Cornish et al., 2008; Ly et al., 2010; Wang et al., 2012). Thus, viomycin inhibits translation by trapping the ribosome in an intermediate state on the translocation pathway and can therefore be considered as a translocation inhibitor, as originally proposed in the 1970′s (Liou and Tanaka, 1976; Modolell and Vazquez, 1977). Importantly, viomycin does not prevent binding of EF-G to the ribosome, nor GTP hydrolysis by EF-G (Modolell and Vazquez, 1977; Peske et al., 2004), however, by blocking translocation viomycin prevents release of EF-G from the ribosome and leads to multiple rounds of futile GTP hydrolysis by EF-G before translocation can occur (Holm et al., 2016). A pre-translocation complex with A/P and P/E hybrid site tRNAs and EF-G trapped by viomycin has been visualized by cryo-electron microscopy (Brilot et al., 2013).

The flipped-out conformations of A1492 and A1493 observed in the presence of viomycin (Figure 6C) or capreomycin (Figure 6D; Stanley et al., 2010) are analogous to those observed in the presence of the misreading inducing 2-deoxystreptamine aminoglycosides (Ogle et al., 2003). However, compared to aminoglycosides, the tuberactinomycin antibiotics induce little, if any, misreading on bacterial ribosomes (Marrero et al., 1980; Akbergenov et al., 2011). Nevertheless, translational misreading (Marrero et al., 1980; Wurmbach and Nierhaus, 1983) and stop codon suppression (Holm et al., 2016) has been reported when using tuberactinomycins in some *in vitro* translation systems. Stabilization of tRNAs in the A-site by viomycin has also been shown to promote back-translocation (Shoji et al., 2006). Viomycin also inhibits mRNA and tRNA release and splitting of ribosomal subunits (Hirokawa et al., 2002; Savelsbergh et al., 2009; Chen et al., 2017) that is normally mediated by RRF and EF-G during ribosome recycling. Additionally, viomycin has been reported to interfere with the canonical translation termination as well as the ArfA-RF2-dependent rescue system (Zeng and Jin, 2016).

Consistent with its binding site, resistance to viomycin results from ribosomes that have mutations or alterations in either the 16S or 23S rRNA (Figure 6E; Yamada et al., 1978; Maus et al., 2005; Johansen et al., 2006), as well as via inactivation of the TlyA methyltransferase that methylates nucleotides C1409 in h44 of the 30S subunit and C1920 in H69 of the 50S subunit (Johansen et al., 2006; Monshupanee et al., 2012). Capreomycin has been shown to disrupt the interaction between *M. tuberculosis* ribosomal proteins uL10 and bL12 (Lin et al., 2014), however, because resistance occurs via mutations in the 23S rRNA, it is likely that this is a secondary effect rather than the prime reason for translation inhibition.

### Odilorhabdins cause miscoding by tethering tRNA to the ribosome

**Figure 7:**
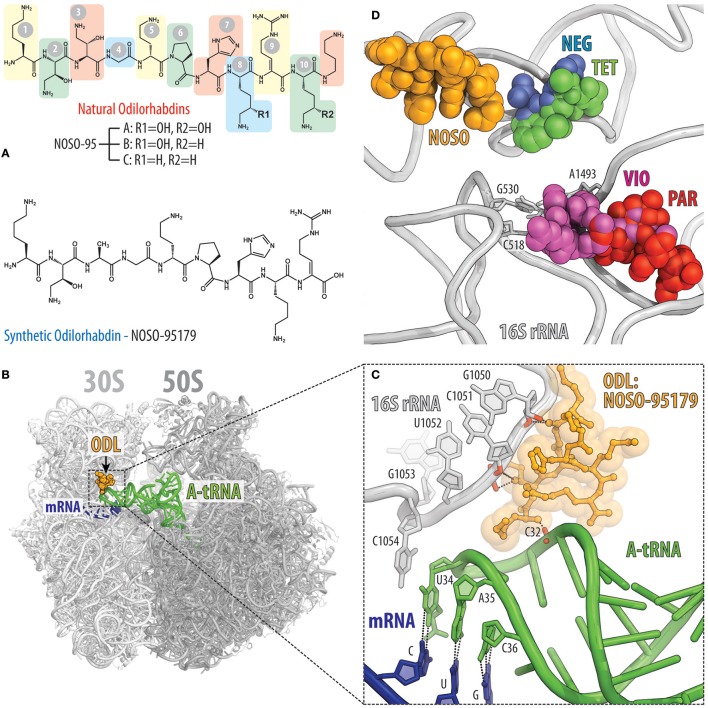
Odilorhabdins bind to the decoding center on the 30S subunit and promote miscoding. **(A)** Chemical structures of natural odilorhabdins NOSO-95A, B, C (top), and the fully-synthetic derivative NOSO-95179 (bottom). **(B)** Overview of the NOSO-95179 binding site (orange) on the *T. thermophilus* 70S ribosome. 30S subunit is light gray, the 50S subunit is dark gray. mRNA is shown in dark blue and A-site tRNAs is displayed in green. **(C)** Close-up view of the NOSO-95179 binding site within the decoding center of the 30S subunit. Shown are interactions of NOSO-95179 with the 16S rRNA and with tRNA. **(D)** Antibiotics that bind in the decoding center on the small ribosomal subunit. Shown are location of the NOSO-95179 binding site relative to the binding sites of other antibiotics known to target the decoding center: paromomycin (PAR, red), viomycin (VIO, magenta), tetracycline (TET, green), negamycin (NEG, blue). Nucleotides of the 16S rRNA that are critical for decoding are shown as sticks.

Recently, a new class of modified peptide antibiotics, odilorhabdins (ODLs), has been discovered (Figure 7A; Pantel et al., 2018). Similarly to tuberactinomycins, ODLs are produced by NRPSs, but from the Gram-negative bacteria *Xenorhabdus nematophila*, which live symbiotically with soil-dwelling nematodes. The first three naturally occurring ODLs were identified by screening the supernatants of 80 cultured *Xenorhabdus* strains for the presence of antimicrobial activity (Pantel et al., 2018). These compounds with molecular weights of 1,296, 1,280, and 1,264 Da were isolated from the supernatant of *X. nematophila* strain K102 and were named NOSO-95A, NOSO-95B, and NOSO-95C, respectively (Figure 7A, top). These ODLs consist of 10 amino acids, including four types of non-proteinogenic amino-acid residues: α,γ-diamino-β-hydroxy butyric acid [Dab(βOH)] in positions 2 and 3; δ-hydroxy lysine (Dhl) in positions 8 and 10; α,β-dehydro arginine (Dha) in position 9; and α,δ-diamino butane (Dbt) in position 11 (Figure 7A, top). The peptidic nature and relative simplicity of ODLs opened the way for improvement of their activity by modifying the chemical structure, resulting in the development of NOSO-95179 (Figure 7A, bottom), a derivative that exhibits better selectivity for bacterial vs. eukaryotic target compared to natural ODLs and thus, represents a preferable lead for further drug development. Overall, ODLs exhibit promising broad-spectrum bactericidal activity and are able to cure bacterial infections in animal models (Pantel et al., 2018).

ODLs bind to the decoding center of the bacterial ribosome at a site not exploited by any other known ribosome-targeting antibiotics (Figures 7B,C). In this binding site, ODLs simultaneously interact with the 16S rRNA as well as with the anticodon loop of the A-site tRNA. Interaction between the ODL and A-site tRNA increases the affinity of the aminoacyl-tRNA to the ribosome, resulting in a decreased accuracy of translation and impeded progression of the ribosome along the mRNA (Figure 7C; Pantel et al., 2018). Although, several classes of antibiotics also target the ribosomal decoding center, the binding site of ODLs is distinct from those of other inhibitors, such as tetracycline and negamycin as well as the tuberactinomycins and aminoglycosides (Figure 7D). Despite this, the overall mechanism of action of ODLs is conceptually similar to that of the aminoglycosides or negamycin, whose mode of translation inhibition depends on the drug concentration. At lower concentrations, these antibiotics induce amino acid misincorporation by reducing the fidelity of decoding, whereas at higher concentrations they interfere with translocation (Wang et al., 2012; Olivier et al., 2014; Polikanov et al., 2014; Pantel et al., 2018). Both activities likely reflect a tighter binding of the tRNA in the A site induced by the ODL. The direct interaction between ODL and tRNA anticodon not only promotes miscoding, but also likely hinders the transition of tRNA from the A site into the P site thus inhibiting translocation at the higher concentrations of the antibiotic.

## Peptide antibiotics targeting the large ribosomal subunit

Binding sites of the majority of peptide antibiotics that target the large 50S subunit cluster around the PTC where peptide bond formation occurs (Figure 2G), for example, streptogramin A (Figure 2H; Hansen et al., 2003; Noeske et al., 2014; Osterman et al., 2017), as well as within the nascent peptide exit tunnel, as seen for the streptogramins B (Figure 2H) and klebsazolicin (Figures 2K; Harms et al., 2004; Noeske et al., 2014; Metelev et al., 2017). The binding sites of the PrAMPs (Figures 2I,J) span from the PTC into the nascent peptide exit tunnel (Graf et al., 2017) and thereby overlap the binding sites of both the streptogramin A and B compounds (Figure 2H). In contrast, the thiopeptide antibiotics, such as thiostrepton, have a distinct binding site from other clinically used antibiotics, which is located far from the PTC and exit tunnel. Instead, the binding site of the thiopeptides is located within the translation factor binding site on the large subunit and encompasses the components of the uL11 stalk base (Figure 2L; Harms et al., 2008).

### Streptogramin antibiotics act synergistically on the large ribosomal subunit

**Figure 8:**
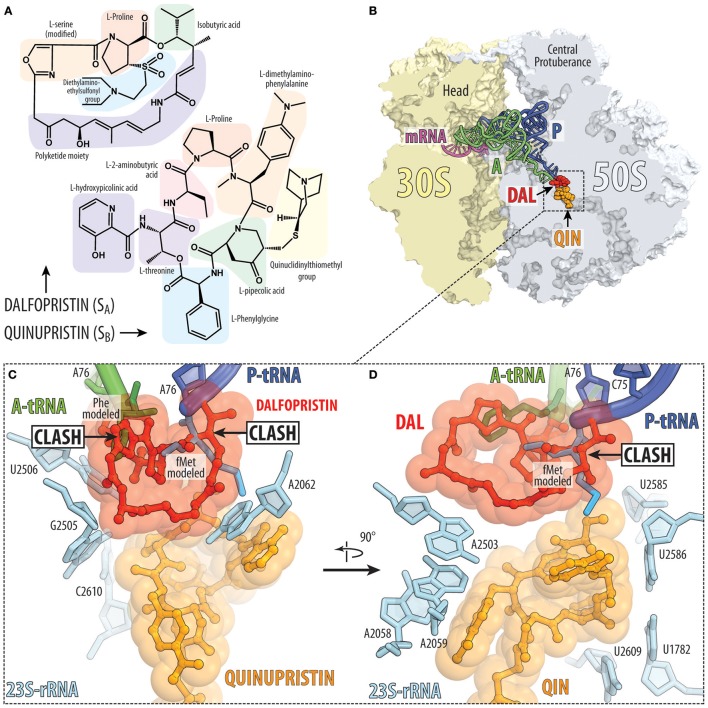
Streptogramins A and B bind within the ribosomal exit tunnel. **(A)** Chemical structures of the streptogramin A (dalfopristin) and B (quinupristin) comprise proteinogenic (e.g., proline, threonine, and serine) as well as non-proteinogenic amino acids, such as phenylglycine and dimethylaminophenylalanine. **(B)** Transverse section of the 70S ribosome revealing the binding site of the streptogramin type A (dalfopristin, DAL, red) and type B (quinupristin, QIN, orange) within the exit tunnel of the large 50S subunit (light blue) (PDB ID 4U26; Noeske et al., 2014). The position of A-tRNA (green) and P-tRNA (blue) as well as mRNA (magenta) on the 30S subunit (yellow) are shown for reference. **(C,D)** Two different views of binding site and interaction of dalfopristin (red) and quinupristin (orange) with 23S rRNA nucleotides (cyan) comprising the PTC and the exit tunnel. The relative position of the aminoacylated CCA-ends of the A-site Phe-tRNA (green) and P-site fMet-tRNA (blue) are shown for reference.

Streptogramin antibiotics are produced by several species of *Streptomyces* and comprise two structurally distinct subclasses: group A, which contain 23-membered macrocyclic polyketide/nonribosomal peptide hybrids and group B, which comprise 19-membered macrocyclic depsipeptides (Figure 8A; Li and Seiple, 2017). Streptogramins have been used as livestock feed additives for decades (Yates and Schaible, 1962) but were not approved by the FDA until the introduction of quinupristin-dalfopristin (Synercid) in 1999. The clinical use of this combination therapy is limited by its intravenous-only formulation and its narrow spectrum of activity, and is reserved for hospitalized patients with multidrug-resistant skin infections or with bacteremia caused by vancomycin-resistant *Enterococcus faecium* (Delgado et al., 2000). An orally bioavailable combination of semisynthetic streptogramins known as NXL-103 (flopristin-linopristin) underwent phase-II clinical trials in Pankuch et al. (2011), but has not progressed further in the clinic.

#### Streptogramin A antibiotics target the peptidyl transferase center

The binding site of group A streptogramins (S_A_) spans the A-site cleft and also encroaches into the P site of the bacterial ribosome (Figures 2G, 8B). Madumycin II (MADU), the simplest S_A_ antibiotic, inhibits the ribosome prior to the first cycle of peptide bond formation (Osterman et al., 2017). It allows binding of the tRNAs to the A and P sites, but prevents correct positioning of their CCA-ends into the PTC, thus making peptide bond formation impossible. Also, binding of MADU induces rearrangement of nucleotides U2506 and U2585 of the 23S rRNA resulting in the formation of the U2506-G2583 wobble base-pair that has been attributed to a catalytically inactive state of the PTC (Schmeing et al., 2005; Osterman et al., 2017). Virginiamycin M is another S_A_ antibiotic that binds in the PTC, causes rearrangements of nucleotides A2062 and U2585 (Hansen et al., 2003; Tu et al., 2005; Noeske et al., 2014) and inhibits binding of A- and P-site substrates (Pestka, 1969; Chinali et al., 1984). In this case, the oxazole ring of virginiamycin M reaches into the A-site cleft, where it establishes hydrophobic interactions.

#### Streptogramin B antibiotics block elongation of the nascent polypeptide chain

The nascent polypeptide exit tunnel of the ribosome is targeted by group B streptogramins (S_B_), such as pristinamycin IA, quinupristin, and virginiamycin S (Figure 8B). Crystal structures of S_A_ (Hansen et al., 2003; Harms et al., 2004; Tu et al., 2005; Noeske et al., 2014; Osterman et al., 2017) and S_B_ (Harms et al., 2004; Tu et al., 2005; Noeske et al., 2014) antibiotics in complex with the ribosome show that both classes bind to adjacent, but not overlapping, sites on the ribosome, which explains their synergistic action (Figure 8B; Vannuffel and Cocito, 1996). As discussed above, S_A_ antibiotics bind at the PTC and prevent proper positioning of the A- and P-site tRNAs (Figures 8C,D), whereas S_B_ antibiotics bind to a site that overlaps with that of macrolides and presumably interfere with the passage of the nascent peptide through the exit tunnel. Interestingly, nucleotide A2062 of the 23S rRNA is sandwiched between the macrocyclic ring of S_A_ compound (for example, dalfopristin) and the S_B_ compound (for example quinupristin) (Figure 8C; Harms et al., 2004; Tu et al., 2005; Noeske et al., 2014), rationalizing why mutation A2062C in the 23S rRNA of *Streptococcus pneumoniae* leads to S_A_ and S_B_ cross-resistance (Depardieu and Courvalin, 2001). Streptogramins have been approved for clinical use, such as Synercid, a mixture of the type A streptogramin dalfopristin and the type B streptogramin quinupristin (Figure 8A; Noeske et al., 2014), which are effective against methicillin-resistant *Staphylococcus aureus* (MRSA; Manzella, 2001).

A unique property of the streptogramin antibiotics is that groups A and B compounds act synergistically *in vivo* and *in vitro*, such that binding of the S_A_ compound promotes the binding of the corresponding S_B_ compound (Parfait et al., 1978). Due to this synergistic action, the concentration of each of the compounds in the mixture required to achieve the inhibitory action is significantly lower than the concentration of each of the compounds when they are used separately (Champney, 2001). The synergistic action also allows streptogramins to overcome some resistance mutations (Vannuffel et al., 1992; Canu and Leclercq, 2001). Moreover, by combining some S_A_ and S_B_ compounds it is possible to convert a bacteriostatic effect into a bactericidal lethality. The basis for the synergy between S_A_ and S_B_ is likely related to a rotation of nucleotide A2062 of the 23S rRNA that was observed upon binding of S_A_ compounds to the PTC (Hansen et al., 2003; Harms et al., 2004; Tu et al., 2005; Noeske et al., 2014; Osterman et al., 2017). In the new drug-induced conformation, A2062 can enhance binding of S_B_ compounds via additional stacking and/or hydrogen bond interactions. Indeed, mutations of A2062 can also lead to streptogramin resistance (Depardieu and Courvalin, 2001). In summary, the action of streptogramins is likely to block both A and P sites, and therefore function during initiation step (Figure 1) or by inducing peptidyl-tRNA drop-off at an early elongation step (Figure 1).

### Proline-rich antimicrobial peptides exhibit distinct mechanisms of action

Unlike most antimicrobial peptides (AMPs), which kill bacteria by disrupting the bacterial membrane, the subclass of proline-rich antimicrobial peptides (PrAMPs) can pass through the bacterial membrane without damaging it and instead inhibit bacterial growth by targeting intracellular processes, such as protein synthesis (Casteels and Tempst, 1994; Mattiuzzo et al., 2007; Seefeldt et al., 2016; Graf et al., 2017). As suggested by their name, PrAMPs are AMPs rich in proline, but also contain many arginine residues. PrAMPs are products of the innate immune system and provide a first line of defense against invading bacteria. To date, PrAMPs have been found in many arthropods, such as insects and crustaceans, as well as in some mammals, such as cows, pigs, goats and sheep (Scocchi et al., 2011; Graf et al., 2017). PrAMPs are usually synthesized as inactive pre-pro-peptides that are matured *via* protease cleavage to yield the active PrAMP peptides. Non-lytic PrAMPs display excellent activity against Gram-negative bacteria, such as *E. coli*, but are generally less active against Gram-positive bacteria, such as *Bacillus subtilis*. This specificity is due to the fact that the uptake of PrAMPs occurs predominantly via the SbmA transporter (Mattiuzzo et al., 2007), but can also utilize the YjiL-MdtM transport system (Krizsan et al., 2015), which are present in most Gram-negative bacteria, but lacking in Gram-positive bacteria. Indeed, resistance to PrAMPs can arise due to deletion or mutation of the SbmA and MdtM transporters (Mattiuzzo et al., 2007; Krizsan et al., 2015; Seefeldt et al., 2015; Florin et al., 2017; Mardirossian et al., 2018). It should be noted that the mammalian PrAMPs, such as Bac7, are generally longer (~60 aa) than insect PrAMPs (~20 aa) and these additional C-terminal residues promote membrane permeabilization (Skerlavaj et al., 1990; Podda et al., 2006), suggesting a dual mode of uptake and action for these PrAMPs.

PrAMPs were shown to interact with the DnaK chaperone, thus initially suggesting that PrAMPs inhibit bacterial growth via interfering with DnaK mediated protein folding (Otvos et al., 2000). Subsequently, it was shown, however, that PrAMPs are equally effective at inhibiting bacterial strains where the gene encoding DnaK was inactivated (Krizsan et al., 2014). This suggested that another intracellular target for PrAMPs must exist. Indeed, PrAMPs were shown to bind to ribosomes and inhibit protein synthesis *in vivo* and *in vitro* (Krizsan et al., 2014; Mardirossian et al., 2014). Despite the diverse array of PrAMPs that have been so far identified, only a subset has been mechanistically investigated. Of the characterized PrAMPs, two distinct mechanisms of action have been identified, both of which involve inhibition of protein synthesis. The oncocin-like PrAMPs or type I PrAMPs allow translation initiation but prevent the transition into the elongation phase (Graf et al., 2017), whereas the apidaecin-like PrAMPs or type II PrAMPs allow translation initiation and elongation but block the translation termination phase (Florin et al., 2017).

#### Type I (oncocin-like) PrAMPs

**Figure 9:**
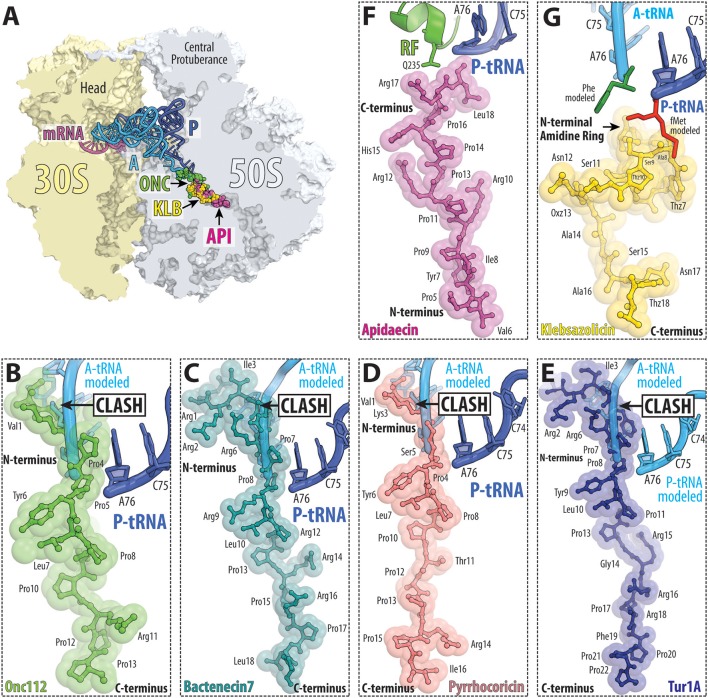
PrAMP and klebsazolicin antibiotics bind within the ribosomal exit tunnel. **(A)** Transverse section of the 70S ribosome revealing the binding site of the PrAMPs oncocin-112 (ONC, green) and apidaecin-137 (API, purple) as well as klebsazolicin (KLB, yellow) within the exit tunnel of the large 50S subunit (light blue). The position of A-tRNA (cyan) and P-tRNA (blue) as well as mRNA (magenta) on the 30S subunit (light yellow) are shown for reference. **(B–G)** Relative binding position of **(B)** oncocin-112 (green, PDB ID 4Z8C; Roy et al., 2015), **(C)** bactenecin-7 (teal, PDB ID 5HAU; Gagnon et al., 2016), **(D)** pyrrhocoricin (light red, PDB ID 5HD1; Gagnon et al., 2016), **(E)** Tur1A (blue, PDB ID 6FKR; Mardirossian et al., 2018), **(F)** apidaecin-137 (magenta, PDB ID 5O2R; Florin et al., 2017) and **(G)** klebsazolicin (yellow, PDB ID 5W4K; Metelev et al., 2017) compared to the CCA-ends of P-site tRNA (blue) and A-site tRNA (cyan) or **(F)** RF1 (green). In panel **(G)**, the A-site Phe and P-site fMet moieties are shown for reference and colored green and red, respectively; THZ, thiazole ring; OXZ, oxazole ring.

The type I PrAMPs encompass both insect and mammalian PrAMPs. One of the best-characterized members is Oncocin and Onc112, which are derivatives of a naturally occurring PrAMP from the milkweed bug *Oncopeltus fasciatus* (Figures 9A,B; Schneider and Dorn, 2001; Knappe et al., 2010, 2011). Other studied type I insect PrAMPs include pyrrhocoricin from the firebeetle *Pyrrhocoris apterus* and metalnikowin-1 from the green shield bug *Palomena prasina*. The best-characterized mammalian type I PrAMP is Bac7(1-16), a C-terminally truncated derivative of the naturally occurring bactenecin-7 (Bac7) PrAMP from the cow (*Bos taurus*). Recently, a type I PrAMP, Tur1A, was also identified from the bottlenose dolphin (*Tursiops truncatus*) (Mardirossian et al., 2018). Structural studies have revealed that type I PrAMPs bind within the ribosomal exit tunnel located on the large subunit (Roy et al., 2015; Seefeldt et al., 2015, 2016; Gagnon et al., 2016; Mardirossian et al., 2018). As expected from the high sequence identity, the insect PrAMPs Onc112, Pyr, Met, mammalian PrAMP Bac7(1-16) and dolphin PrAMP Tur1A bind with similar extended conformations within the exit tunnel (Figures 9B–E). The orientation of type I PrAMPs is inverted with respect to a nascent polypeptide chain, such that the N-terminus is located at the peptidyl transferase center (PTC) and the C-terminus extends into the ribosomal tunnel. Mutations of 23S rRNA nucleotides located within the ribosomal tunnel, such as A2503C, A2059C, and especially the A2503C/A2059C double mutation lead to increased resistance to Onc112, but surprisingly not to Bac7 (Gagnon et al., 2016). Consistent with biochemical studies (Seefeldt et al., 2015, 2016; Gagnon et al., 2016), the structures reveal that the type I PrAMPs do not significantly overlap with the binding site of a P-site tRNA and thus allow translation initiation to occur uninhibited (Roy et al., 2015; Seefeldt et al., 2015, 2016; Gagnon et al., 2016). By contrast, the N-terminal region of the type I PrAMPs sterically overlaps the binding site of the CCA-end of an A-site tRNA. This suggests that type I PrAMPs prevent the transition from initiation to elongation by blocking the binding and accommodation of the aa-tRNA at the PTC on the large subunit (Graf et al., 2017).

#### Type II (apidaecin-like) PrAMPs

The type II PrAMPs so far include only the insect PrAMPs belonging to the apidaecin subfamily. The best-characterized member is Api137, a derivative of the naturally occurring PrAMP apidaecin 1b from the honey bee *Apis mellifera*. Apideacin-like PrAMPs are also found in other bees, wasps and hornets. Structural studies revealed that similar to type I PrAMPs, type II PrAMPs, such as Api137, also bind within the ribosomal exit tunnel (Figures 9A,F; Florin et al., 2017). However, the orientation of the type II PrAMPs is inverted with respect to type I PrAMPs, i.e., type II PrAMPs have the same orientation as a nascent polypeptide chain with the C-terminus located at the peptidyl transferase center (PTC) and the N-terminus extending down the ribosomal tunnel (Figure 9F). Biochemical studies show that Api137 does not inhibit translation initiation or elongation despite the overlap in binding site with the growing nascent polypeptide chain (Figure 9F). This paradox was resolved by the finding that Api137 has a very low affinity for empty ribosomes and require the presence of a termination release factor, RF1 or RF2, for stable binding (Florin et al., 2017). Presumably, the low affinity of Api137 in the absence of RF1/RF2 leads to its dissociation via prolongation of the nascent chain during translation elongation. During termination, Api137 does not interfere with binding of RF1/RF2 to the termination ribosome, nor with peptidyl-tRNA hydrolysis and release of the polypeptide by RF1/RF2. In fact, release of the polypeptide is a pre-requisite to allow Api137 to enter the ribosomal tunnel and engage its binding site. Following peptide release, however, binding of Api137 to the ribosome traps RF1/RF2 on the ribosome, even in the presence of RF3 (Florin et al., 2017). Thus, the action of Api137 needs to occur in the short time window following RF1/RF2-mediated peptide release, but before dissociation of RF1/RF2 from the ribosome.

Although the binding site of Api137 overlaps with type I PrAMPs, one major difference is that Api137 does not block entry of the A-site tRNA into the PTC. Instead, the C-terminus of the Api137 is positioned such that direct contact with RF1 and RF2 in the A site can occur. Specifically, Arg17 of Api137 can form direct hydrogen bond (H-bond) interactions with the sidechain of glutamine 235 (Q235) of the conserved GGQ motif. This is consistent with biochemical findings showing that mutations of Arg17 in Api137 decrease the ribosome affinity and reduce its inhibitory properties (Krizsan et al., 2014). In addition, the C-terminal carboxylate group of Api137 is within H-bond distance to the ribose hydroxyl of A76 of the deacylated P-site tRNA (Figure 9F), which could also contribute to trapping RF1/RF2 by preventing the ribosome from undergoing the RF3-stimulated transition into the rotated state required for RF1 or RF2 dissociation. Mutations in RF1 and RF2 as well as in ribosomal protein uL3 have been identified that confer resistance to Api137 (Florin et al., 2017). These mutations sites are located distant from the Api137 binding site and are therefore likely to confer resistance by altering RF1/RF2 binding such that dissociation can occur even in the presence of Api137. Additionally, mutations within ribosomal tunnel can also confer resistance to Api137, including 23S rRNA mutations A2059C and A2503G, as identified for type I PrAMPs, such as Onc112, but also alterations within ribosomal proteins uL4 and uL22 rendered cells resistance to Api137 (Florin et al., 2017).

It should be noted that the number of ribosomes within a bacterial cell, such as *E. coli*, is much greater than the number of RF1 and RF2 molecules (by 200- and 25-fold, respectively; Bremer and Dennis, 1996; Schmidt et al., 2016) and therefore, Api137 can only trap RF1 and RF2 on a small subset of the available ribosomes. Nevertheless, this leads to a rapid depletion of the free pools of RF1 and RF2 in the cell, such that the vast majority of ribosomes become stalled during translation termination. Because of the absence of RF1 and RF2, an increased level of stop codon readthrough is observed on the termination stalled ribosomes. Surprisingly, the stop codon readthrough induced by Api137 is considerably higher than that induced by the classical misreading antibiotic streptomycin (Florin et al., 2017). Thus, in summary, type II PrAMPs such as Api137, have a dual mode of action to, on one hand, trap RF1 and RF2 on a minority of ribosomes within the cell and, on the other hand, to stall the majority of ribosomes at the termination phase due to the absence of available RFs, which in turn increases the rates of stop codon readthrough.

### Klebsazolicin obstructs the ribosomal exit tunnel

Klebsazolicin (KLB) is the first member of a new class of protein synthesis inhibitors, which comes from the opportunistic human pathogen *Klebsiella pneumonia*, and was discovered recently using a genome mining approach (Metelev et al., 2017). This method allows one to harness a much greater chemical diversity and can result in the discovery of entirely new molecular scaffolds. Analysis of genomic data makes it possible to identify gene clusters encoding biosynthetic pathways for potential drug candidates, which may otherwise escape attention due to their inactivity under laboratory growth conditions (Doroghazi et al., 2014). Ribosomally-synthesized post-translationally modified peptides (RiPPs) are among the most abundant antimicrobial agents synthesized by various bacteria, including human microbiota (Donia et al., 2014; Donia and Fischbach, 2015).

KLB is the first linear azole-containing RiPP for which the mode of binding to its target, the bacterial ribosome (Figures 9A,G), has been structurally characterized. KLB is synthesized on the ribosome as a precursor, which undergoes post-translational modifications by dedicated enzymes encoded in a compact gene cluster (Metelev et al., 2017). KLB appears to have a modular structure: its 14 N-terminal residues are essential for the inhibition of the ribosome, while its nine C-terminal residues are likely to be important for the uptake of the molecule and are not essential for ribosome binding (Metelev et al., 2017; Travin et al., 2018). It is likely that natural sensitivity/resistance to KLB is determined not by the differences in the ribosome target, but rather to differences in uptake. Moreover, KLB can be expressed in a surrogate *E. coli* host (Metelev et al., 2017), which suggests avenues for future rational drug design: by changing the primary sequence of amino acids in the KLB precursor, it is possible to change properties of the final processed compound.

Structural analysis of the ribosome-KLB complex reveals that the compound binds in the nascent peptide exit tunnel to a site that overlaps with the binding sites of macrolides, S_B_, the PrAMPs, and significantly obstructs the tunnel (Figure 9G; Metelev et al., 2017). Similar to PrAMPs, KLB interacts with the ribosome mainly via stacking with rRNA bases. However, unlike PrAMPs, which bind the ribosome in an elongated conformation (Figures 9B–F), KLB adopts a compact, globular conformation within the exit tunnel (Figure 9G). KLB inhibits protein synthesis by blocking the elongation after only three amino acids of the nascent peptide have been polymerized (Metelev et al., 2017). The KLB binding site does not overlap with the aminoacyl moieties of the A- and P-site tRNAs (Figure 9G) and the main occlusion point occurs around the macrolide binding site leaving some space between the PTC active site and the bound KLB molecule, so that the 2–3 amino acid long nascent peptide can fit. A unique and essential 6-membered amidine ring of KLB forms multiple interactions with the universally conserved nucleotides of the 23S rRNA at the heart of the PTC. For example, it forms two H-bonds with U2584, which resembles a non-canonical U-U base-pair.

### Thiopeptide antibiotics that interfere with translation factor binding

**Figure 10:**
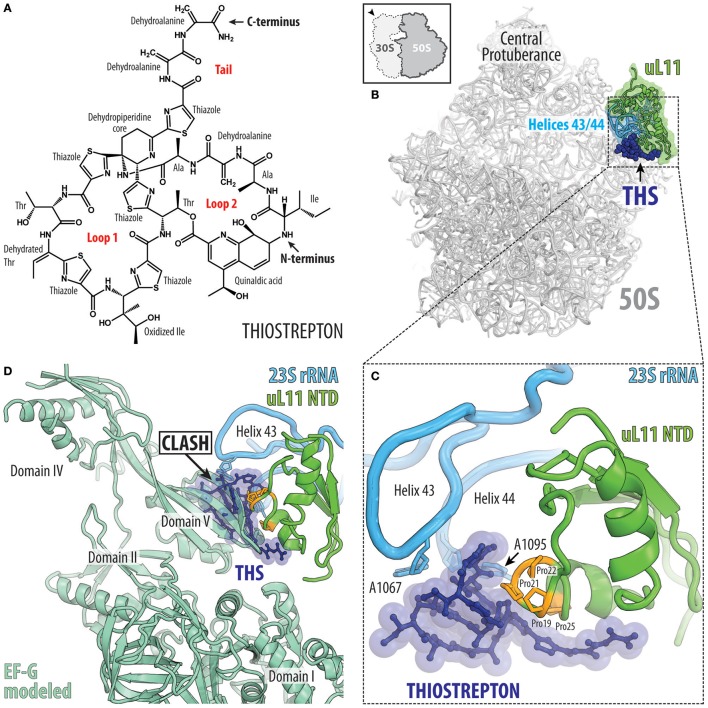
Thiostrepton binding site on the large ribosomal subunit. **(A)** Chemical structure of the thiostrepton. **(B)** The binding site of the thiostrepton (THS, blue) on the large 50S subunit of *Dienococcus radiodurans* (gray) (PDB ID 3CF5; Harms et al., 2008). The position of 23S rRNA helices H43 and H44 (cyan) and ribosomal protein uL11 (green) are shown for reference. The 50S subunit is viewed from the subunit interface as indicated by the inset at the top left. **(C)** Close-up view of the thiostrepton binding site showing its interactions with 23S rRNA nucleotides A1065 and A1095 located at the tips of helices H43 and H44 (cyan) as well as proline residues (orange) within the N-terminal domain (NTD) of ribosomal protein uL11 (green). **(D)** Overlap in binding position of thiostrepton (THS, blue) and domain V of EF-G (pale green).

There are an array of different thiopeptide antibiotics that inhibit translation either by interacting with translation factor EF-Tu, for example, GE2270A, or by directly binding to the ribosome, with the best characterized being thiostrepton, nosiheptide, and micrococcin (Bagley et al., 2005; Nicolaou et al., 2009). These antibiotics are synthesized as precursor polypeptides by the ribosome and then are post-translationally modified to yield the active compound (Wieland Brown et al., 2009). The chemical structure of thiostrepton can be separated into two loops (loop1 and 2) and a dehydroalanine tail, which are linked together by a tetrahydro-pyridin-3-ylamine moiety (Figure 10A). The 16 distinct chemical moieties that comprise thiostrepton include many thiazole rings as well as non-canonical and canonical amino acids, including for example, threonine, isoleucine, alanine and dehydroalanine (Figure 10A; Kelly et al., 2009). The thiopeptide antibiotics are effective against Gram-positive bacteria, in particular, methicillin-resistant *Staphlococcus aureus* (MRSA), as well as against the malarial parasite *Plasmodium falciparum* (Aminake et al., 2011), but suffer from low water solubility and poor bioavailability, which has precluded their use in human medicine (Wilson, 2009).

The crystal structure of the *Deinococcus radiodurans* large subunit bound to thiostrepton (as well as nosiheptide and micrococcin; Harms et al., 2008) revealed that this class of antibiotics bind in a cleft formed between the N-terminal domain (NTD) of ribosomal protein uL11 and helices H43 and H44 of the 23S rRNA (Figure 10B). The solution NMR structure of thiostrepton compares well with the X-ray structure and reveals high flexibility of the dehydroalanine tail (Jonker et al., 2011). Within the cleft, thiostrepton interacts with nucleotides A1067 and A1095, located at the tips of H43 and H44, respectively, and the thiazole rings of thiostrepton stack upon the proline residues located in the NTD of uL11 (Figure 10C). This thiopeptide binding site is distinct when compared to other clinically used antibiotics and therefore cross-resistance with thiopeptides has not been reported. Nevertheless, mutations in A1067, A1095, or in the numerous proline residues of the uL11-NTD reduce thiopeptide binding and confer drug-resistance in bacteria and archaea (Wilson, 2009; Baumann et al., 2010). Furthermore, the producer of thiostrepton, *Streptomyces azureus*, inhibits drug binding to its own rRNA by 2′-O-methylation of position A1067 (Thompson et al., 1982). Eukaryotic 80S ribosomes are naturally resistant to thiostrepton, most probably due to sequence differences in uL11, which is in agreement with the observations that yeast 80S ribosomes bearing bacterial uL11 are sensitive to the drug (Garcia-Marcos et al., 2007). The thiopeptide binding site on the large subunit sterically overlaps with the binding site of translation factors, such as the IF2, EF-Tu and EF-G (Figure 10D; Harms et al., 2008). Consistently, thiostrepton has been reported to inhibit IF2-dependent initiation complex formation (Brandi et al., 2004; Grigoriadou et al., 2007), EF-Tu delivery of the aa-tRNA to the A-site (Gonzalez et al., 2007) as well as accommodation of EF-G, which leads to inhibition of the translocation reaction (Rodnina et al., 1999; Seo et al., 2006; Pan et al., 2007; Mikolajka et al., 2011; Walter et al., 2012).

## Concluding remarks

**Figure 11:**
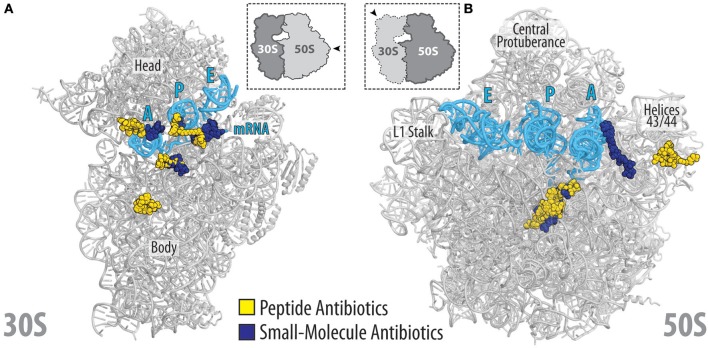
Relative location of peptide and small-molecular antibiotics on the bacterial ribosome. **(A)** Overview of the binding sites of the peptide (yellow) and small-molecular (blue) antibiotics targeting the small (30S) ribosomal subunit: edeine B, GE81112, dityromycin, viomycin, odilorhabdin, negamycin, tetracycline, paromomycin, streptomycin, spectinomycin, amicoumacin, pactamycin, kasugamycin. **(B)** Overview of the binding sites of the peptide (yellow) and small-molecular (blue) antibiotics targeting the large (50S) ribosomal subunit: streptogramin type A (dalfoprsitin) and type B (quinupristin), oncocin-112, apidaecin-137, klebsazolicin, thiostrepton, orthosomycin (avilamycin), macrolides (erythromycin, carbomycin, spiramycin, tylosin), chloramphenicol, hygromycin A, A201A, lincosamides (clindamycin), oxazolidinones (linezolid). The relative position of A, P, and E-site tRNAs (cyan) are shown, and 23S rRNA helices H43/44 is highlighted for reference.

The available structures of peptide antibiotics on the ribosome illustrate the diverse manners in which these inhibitors interact with the ribosome and interfere with translation. High-resolution structures now open the way for structure-based design to develop peptide antibiotics with improved properties by identifying sites that can be modified to enable additional interaction with the ribosome. Similarly, the structures also identify residues that are not critical for ribosome binding and therefore can be utilized to optimize parameters such as uptake and retention, serum stability as well as reduced membrane permeabilization and toxicity. The proximity of the binding sites of peptide antibiotics on the ribosome in relation to other classes of ribosome-targeting antibiotics (Figures 11A,B) also offers the opportunity to generate hybrid compounds that span over multiple binding sites. The increase in sequenced genomes coupled with improved data mining algorithms is leading to the identification of potential gene clusters encoding biosynthetic pathways for novel peptide antibiotics and PrAMPs. It will be interesting to see what novel ribosome-targeting peptide antibiotics these approaches will yield and to investigate their binding sites on the ribosome and mechanism of action to inhibit translation.

## Author contributions

YP and NA: prepared the figures; DW, BB, NA, and YP: wrote the manuscript.

### Conflict of interest statement

The authors declare that the research was conducted in the absence of any commercial or financial relationships that could be construed as a potential conflict of interest.
